# Complementarity of Binding Motifs is a General Property of HLA-A and HLA-B Molecules and Does Not Seem to Effect HLA Haplotype Composition

**DOI:** 10.3389/fimmu.2013.00374

**Published:** 2013-11-14

**Authors:** Xiangyu Rao, Rob J. De Boer, Debbie van Baarle, Martin Maiers, Can Kesmir

**Affiliations:** ^1^Theoretical Biology and Bioinformatics, Utrecht University, Utrecht, Netherlands; ^2^Immunology Department, Wilhelmina Children’s Hospital, University Medical Center Utrecht, Utrecht, Netherlands; ^3^National Marrow Donor Program, Minneapolis, MN, USA

**Keywords:** haplotypes, HLA antigens, selection, genetic, peptide binding, bioinformatics, computational biology

## Abstract

Different human leukocyte antigen (HLA) haplotypes (i.e., the specific combinations of HLA-A, -B, -DR alleles inherited together from one parent) are observed in different frequencies in human populations. Some haplotypes, like HLA-A1-B8, are very frequent, reaching up to 10% in the Caucasian population, while others are very rare. Numerous studies have identified associations between HLA haplotypes and diseases, and differences in haplotype frequencies can in part be explained by these associations: the stronger the association with a severe (autoimmune) disease, the lower the expected HLA haplotype frequency. The peptide repertoires of the HLA molecules composing a haplotype can also influence the frequency of a haplotype. For example, it would seem advantageous to have HLA molecules with non-overlapping binding specificities within a haplotype, as individuals expressing such an haplotype would present a diverse set of peptides from viruses and pathogenic bacteria on the cell surface. To test this hypothesis, we collect the proteome data from a set of common viruses, and estimate the total ligand repertoire of HLA class I haplotypes (HLA-A-B) using *in silico* predictions. We compare the size of these repertoires to the HLA haplotype frequencies reported in the National Marrow Donor Program (NMDP). We find that in most HLA-A and HLA-B pairs have fairly distinct binding motifs, and that the observed haplotypes do not contain HLA-A and -B molecules with more distinct binding motifs than random HLA-A and HLA-B pairs. In addition, the population frequency of a haplotype is not correlated to the distinctness of its HLA-A and HLA-B peptide binding motifs. These results suggest that there is a not a strong selection pressure on the haplotype level favoring haplotypes having HLA molecules with distinct binding motifs, which would result the largest possible presented peptide repertoires in the context of infectious diseases.

## Introduction

The human leukocyte antigen (HLA) genes are the most polymorphic coding loci known in humans. The HLA gene cluster is located on the major histocompatibility complex (MHC) on chromosome 6, and contains over 200 genes. The two groups of loci that contain the MHC class I and II genes dictating T cell responses are the most polymorphic. It is widely accepted that this variability is maintained by balancing selection, as individuals that are heterozygous in their HLA class I and II loci seem to have a better outcome in infections diseases [see e.g., for HIV-1 (1)]. In line with this, it has been demonstrated that in several species, and up to a certain extent in humans, females prefer to mate with males having a dissimilar HLA to increase the chance of their offspring to survive infectious diseases (2, 3). We have argued that the heterozygous advantage is on its own not enough to maintain such a large degree of polymorphism, and that the frequency dependent co-evolution with pathogens should also play a major role (4).

On the functional level the HLA polymorphism seems to be much smaller. It is well established that MHC class II molecules have largely overlapping peptide repertoires [see e.g., (5)]. In the last few years we and others showed this to be also true for class I alleles (6–9). This promiscuous peptide binding is not limited to the alleles within each locus, and extends to alleles from different MHC class I loci (7, 9). This property has important evolutionary consequences as heterozygous individuals carrying genetically different HLA molecules that nevertheless have overlapping peptide repertoires should have a diminished heterozygous advantage.

Recombinations occur frequently in the HLA region and they generate novel HLA haplotypes (10). However, the number of haplotypes found in human populations is far lower than the number of alleles observed (11), suggesting that not all the HLA haplotypes have the same chance of becoming established in human populations. Indeed, different haplotypes occur with very different frequencies in different ethnic groups/subpopulations^1^ ^,^^2^. The “usefulness” of HLA molecules is expected to play a role in determining the haplotype frequencies: haplotypes carrying HLA molecules that are protective for certain diseases are expected to be present in relatively high frequencies in endemic areas. Indeed, several HLA haplotype disease associations have been established, e.g., in autoimmune diseases (12), squamous cell cervical cancer (13) and recurrent aphthous stomatitis (14). Along these lines, one can speculate that it should be advantageous to have MHC molecules with distinct binding specificities in a haplotype, because such haplotypes would provide an individual with more epitopes eliciting T cell responses during an infectious disease than haplotypes containing HLA molecules with similar binding motifs. This hypothesis is rather impossible to test experimentally, because one needs to determine the total set of peptides presented by a very large set of HLA molecules in cells infected by different viruses. Such experiments (e.g., eluting peptides from HLA molecules expressed by a cell) are typically done at small scales because they are time consuming. Therefore, we here study the peptide repertoires of HLA molecules that are estimated to be combined in a haplotype (i.e., the ones with a strong linkage disequilibrium) using an *in silico* approach. This approach unfortunately suffers of two main limitations. First, the quality of the peptide-HLA predictions differs between the loci. The quality of HLA-A and HLA-B peptide binding predictions is very reliable. For the other loci (HLA-C, HLA-DR, etc.) the predictions are of low quality (15), and are therefore left out of this analysis. Second, having distinct HLA binding motifs might also be important in the context of cancer or autoimmunity, however, it is very complicated to determine the role of HLA haplotypes in these diseases. Therefore, we perform our analysis explicitly for infectious diseases, and focus on the peptides that can be presented from common viruses only. Still, we think that our study is a first step to investigate the role of HLA binding motifs in the evolution of HLA haplotype frequencies.

## Materials and Methods

### NMDP data

All estimated HLA-A-B haplotype frequencies were downloaded from the National Marrow Donor Program (NMDP) database, which was established to develop and maintain a registry of HLA-typed volunteer unrelated donors for patients requiring a hematopoietic stem cell transplant (16)^3^. Four predominant US ethnic and racial groups were included in this data set: European Americans, African Americans, Asians, and Hispanic (17). Haplotype frequencies were estimated separately for each ethnic group using an implementation of the expectation maximization (EM) algorithm (18, 19).

The linkage disequilibrium, *D*, between two alleles in each haplotype was expressed as the difference between the observed and expected haplotype frequency: *D* = *f_AB_*_−_*f_A_f_B_*, where *f_AB_* is the observed (estimated) haplotype frequency, *f_A_* is the allele frequency of the HLA-A molecule in the haplotype and *f_B_* is the allele frequency of the HLA-B molecule in this haplotype. *D* is easy to calculate, but has the disadvantage of depending on the frequency of the alleles. In order to overcome this drawback, the normalized measure, D′, was calculated a $D\prime =\frac{D}{D_{\max}},$ where *D*_max_ is the lesser of *f_AB_* or (1 − *f_A_*) (1 − *f_B_*) when *D* < 0 and is the lesser of *f_A_*(1 − *f_B_*) or *f_B_*(1 − *f_A_*) when *D* > 0. The advantage of this measure of disequilibrium is that it ranges between −1 and 1, regardless of the allelic frequencies in the sample. $\left|D\prime \right|=1$ indicates complete LD and *D*′ = 0 corresponds to total absence of LD.

Linkage disequilibrium statistics were calculated for each haplotype to identify the haplotypes that have a significantly positive *D*′.

### HLA ligand prediction

To be able to perform our analysis for as many as possible HLA molecules, we used NetMHCpan (15) to predict peptide-HLA binding affinity. NetMHCpan assigns to each peptide-HLA pair a predicted IC50 value, indicative of the predicted binding affinity. To assess whether a peptide binds to an HLA molecule depends on the choice of binding threshold, and the optimal threshold has been discussed (20). If one assumes that all HLA molecules use a fixed threshold, one can use the default threshold of 500 nM (21, 22), otherwise a 5000 nM threshold can be used to allow for the comparison of more weakly binding peptides. However, using a fixed threshold to define predicted binders result in large differences in the predicted repertoire sizes between HLA molecules. For instance using a fixed threshold of 500 nM, the HLA repertoire sizes range between 20 and 6574 peptides for the viral set listed in Table S2 in Supplementary Material. As such a variance could introduce large biases in our analysis, we defined the 1% top-ranking peptides as candidate binders for each HLA molecule. This gives each HLA molecule the same ligand repertoire size (i.e., 570 binders per HLA molecule for the viral set listed in Table S2 in Supplementary Material). To check the consistency of our results with respect to these parameters, we repeated every analysis with the fixed threshold of 500 nM. All results presented below were derived using the threshold of 1% to define candidate binders, and remain similar for a fixed threshold, unless mentioned otherwise. To test whether our results change if one were to use a much larger data set, we also generated predicted binders using a much larger set of viruses (see below), in which case each HLA molecule had 60-fold more binders.

### Viral data

The proteomes of 17 common human viruses were downloaded from the European Bioinformatics Institute website^4^ (downloads were made in October 2006, listed in Table S2 in Supplementary Material) as the source of potential HLA ligands. To extend this data set, we downloaded another set of proteomes (downloads were made in October 2008)^4^ from viruses that are known to infect mammalians (*n* = 904). We used the HLA-peptide binding predictors (see above) to screen all possible unique virus-derived 9-mer peptides.

### Peptide repertoire overlap

We define the peptide repertoire overlap between two HLA alleles in the same HLA-A-B haplotype, *F_p_*, as the fraction of overlapping ligands between these two HLA class I molecules among all of their ligands: $F_{p}=\frac{A⋂B}{A⋃B}$, where *A* and *B* are the ligand sets for HLA-A and -B molecule, respectively. *F_p_* = 1 implies that the HLA-A and HLA-B molecules belonging to the same haplotype have the same epitope repertories while *F_p_* = 0 indicates completely different peptide repertoires for two HLA molecules.

## Results and Discussion

### Determining HLA-A-B haplotypes

The “true” HLA haplotypes can be determined either by molecular haplotyping or family-based segregation studies (23–25). However, both approaches are expensive and laborious, and therefore, statistical methods are typically used to infer haplotypes from datasets covering large population of individuals with known HLA genotypes (26). Several methods have been proposed to infer HLA haplotypes from genotype data, and in recent studies the performance of two most commonly used approaches, EM algorithm based (implemented in Arlequin V3.0), and the Bayesian algorithm based (implemented in PHASE V2.1.1), have been compared (27, 28). Unfortunately, neither of the methods could infer all of the known haplotypes: incorrect haplotypes were estimated in more than 30% of the cases. However, once the sample size increases, the power of these statistical methods is expected to increase tremendously.

National marrow donor program^2^ provides, to our knowledge, the largest repository of HLA-typed donors. Here use of statistical methods should become more reliable (16): for the HLA-A-B haplotype, the total chromosome counts (2N) for the four major ethnic groups exceeds 2000. On the NMDP webpage^3^, the high-resolution allele and haplotype frequencies [estimated by EM method, (18, 19)] are available (17). Focusing on HLA-A-B haplotypes, the most common haplotypes found in US population (separated into four main ethnic groups) are summarized in Table 1 (adopted from bioinformatics.nmdp.org, December 2007 version). Alternatively Allele frequencies web server^1^, provides allele frequencies established in smaller, but probably better defined studies (29).

**Table 1:** **Occurrences of the three most common HLA-A-B frequency ranked haplotypes in four major ethnic groups in US (adopted from bioinformatics.nmdp.org)**.

		EUR		AFA		API		HIS	
HLA-A	HLA-B	*F* (%)	Rank	*F* (%)	Rank	*F* (%)	Rank	*F* (%)	Rank
0101	0801	9.55	1	1.50	6	0.41	46	2.21	2
0201	4402	5.70	3	1.33	9	0.17	130	1.94	4
0201	4501	0.05	200	1.66	3	–	–	0.23	105
0201	5101	2.00	9	0.61	26	0.91	25	2.20	3
0207	4601	–	–	–	–	3.34	2	–	–
0301	0702	6.01	2	1.73	2	0.26	82	1.92	5
2902	4403	2.38	7	1.08	15	0.03	433	2.54	1
3001	4201	–	–	2.96	1	–	–	0.40	50
3303	4403	–	–	0.09	261	2.94	3	0.16	156
3303	5801	0.08	162	0.28	88	4.53	1	0.10	230

EUR, Caucasian; AFA, African; API, Asian; HIS, Hispanic. *F* stands for population frequency in percentages.

In the NMDP database 660 possible haplotypes are reported for European Americans. However, many of these haplotypes are bound to be falsely predicted, e.g., due to the limited number of individuals carrying particular combinations of specific HLA molecules. To decrease the amount of wrongly identified haplotypes in our analysis, we apply a rather strict criterion for considering a predicted haplotype as a “true” haplotype: we demand a positive LD value that is significantly different than zero (*p* < 0.01, see Materials and Methods). This criterion decreases the number of haplotypes to 60 for European Americans. These 60 haplotypes are estimated to cover 58% of the population (see Table 2), i.e., current statistical methods and data sets (even the large repositories like NMDP) remain rather limited in providing the HLA haplotype diversity of a population. For other ethnical groups, the number of reliable haplotypes drops to 30–40 per ethnic group, even though the number of possible haplotypes was the same or higher (see Table 2 and footnote text 3). The population coverage in non-European groups was lower, 30–40% of their respective populations, possibly due to the lower number of individuals with known HLA-typing. All together we detected 120 reliable unique haplotypes by summing over these ethnical groups (the full list of haplotypes can be found in Table S1 in Supplementary Material).

**Table 2:** **Numbers of different haplotypes with a significantly positive LD in four major US ethnic groups**.

Ethnicity	Haplotype # (%)
EUR	60 (57.7)
API	34 (35.4)
HIS	43 (33.5)
AFA	43 (33.7)

Population coverage in percentages is given within parenthesis.

### Peptide repertoire of an haplotype

Having identified the HLA-A-B haplotypes for the US population, we next estimated the overlaps between peptide repertoires of HLA-A and -B molecules that belong to the same haplotype.

We used an *in silico* approach and predicted the peptide repertoire of all HLA-A and HLA-B alleles that are part of the 120 predicted haplotypes, using the proteomes of common viruses (see Table S2 in Supplementary Material) and HLA-peptide binding predictor NetMHCpan (15, 30) (see Materials and Methods). NetMHCpan is the only prediction system available right now that can reliably predict the peptide binding affinities for the large set of HLA-A and HLA-B molecules we are taking into account in this study. The analysis of the 120 significant haplotypes demands predictions for 39 HLA-A and 63 HLA-B molecules. This predictor assigns an IC50 value to each peptide-HLA pair, which can be used as a predicted binding affinity. Using the widely accepted IC50 value of 500 nM as a threshold to distinguish binders from non-binders, generated a large variation in the predicted repertoire sizes of different HLA molecules (20–6574 peptides for the viral set listed in Table S2 in Supplementary Material), which could strongly bias our results. As the physiologically relevant IC50 values are difficult to estimate for each HLA molecule, we have chosen a simplified approach and define the peptide repertoire as the top 1% peptides with the highest HLA binding affinities for each HLA molecule. This approach removes the potential bias introduced by different repertoire sizes, but ignores the fact that some HLA molecules can be more specific than others, and therefore present much fewer peptides.

**Figure 1:**
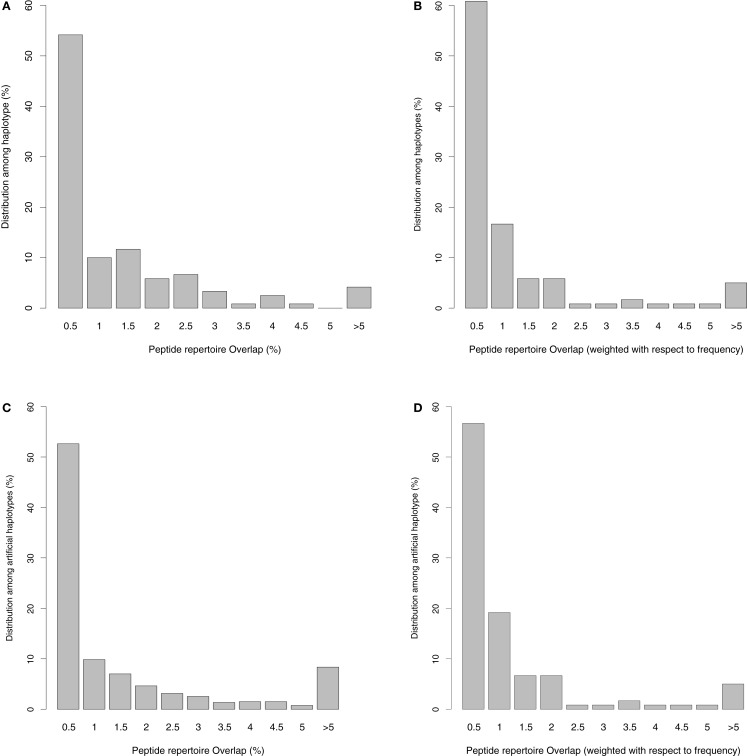
**Distribution of the presented peptide overlap (given in percentages, see Materials and Methods) between HLA-A and HLA-B molecules belonging to the same haplotype**. **(A)** The distribution of the peptide overlaps for the 120 unique haplotypes we identified in the US population. **(B)** The distribution in A is weighted with the frequency of the haplotype in the population. If a haplotype was found in more than one ethnicity in US, we have taken the maximum frequency into account, normalizing the frequencies such that the sum remains one. In **(C)** we plot the overlaps found in artificially generated haplotypes (created by reshuffling the HLA-A and -B molecules from the 120 unique haplotypes). This plot is representative of 100 sets of artificially generated haplotypes. **(D)** The weighted peptide overlaps for the artificial haplotypes, where we estimate the frequency of the haplotype as the multiplication of the frequency of HLA-A and HLA-B molecule in the population (normalized to let the sum of the frequencies of the artificial haplotypes remain one).

The unique haplotypes listed in Table S1 in Supplementary Material have an average peptide overlap of 1.1% (with a standard deviation of 1.8%, and a median of 0.44%) using top 1% threshold. The distribution of the overlaps is given in Figure 1A, and varies somewhat among the haplotypes. Out of 120 haplotypes, 36 (30%) have non-overlapping peptide repertoires, at least for the common viruses that we tested (see Table S1 in Supplementary Material). Only for five haplotypes is the repertoire overlap higher than 5%, and HLA-A0101-B1517 with an overlap of 11.8% is the highest. HLA-A0101-B1517 is a rare haplotype and occurs only in Asian Americans with a population frequency of 0.4%. To test whether or not rare haplotypes tend to have larger overlaps than common haplotypes, we weighted the overlaps found in Figure 1A with the population frequency of the HLA-A-B haplotypes (Figure 1B). Since the weighted overlaps remain very similar to the unweighted overlaps (Figures 1A,B), there is no evidence for a trend of rare haplotypes having the largest overlaps. In line with this, the frequency of a haplotype is only weakly correlated with the degree of peptide overlap between the haplotype’s HLA-A and HLA-B molecules (*r* = −0.08, *p* = 0.4, Spearman rank correlation). Apparently, there is no selection pressure increasing the frequency of the haplotypes with a small peptide repertoire overlap.

These results were obtained using the top 1% peptides with the highest HLA binding affinities as the set of presented peptides per molecule. Using the set of common viruses listed in Table S2 in Supplementary Material, this threshold results in approximately 570 predicted binders per HLA molecule. To test whether the results presented in Figure 1A would be sensitive to the number of peptides, we collected proteomes for a much larger set of mammalian viruses (*n* = 904), which contains approximately three million unique peptides of nine amino acids. Using the same 1% threshold for this larger set, we predict for each HLA molecule the extended peptide repertoire and calculate the overlaps as explained before. The distribution of the overlaps hardly changes despite the fact that we enlarged the presented peptide repertoire 60-fold per molecule (see Figure S1 in Supplementary Material). As the results presented in Figure 1A seem fairly insensitive to the number of peptides used, we perform the rest of the analysis on the small data set of common viruses.

To test whether or not HLA binding motifs affect haplotype compositions requires comparison of the peptide overlaps of “true” haplotypes with those of “random” haplotypes. To do this, we reshuffled HLA-A and HLA-B molecules in the 120 “true” haplotypes to calculate an expected peptide repertoire overlap for randomly made haplotypes. Although the HLA molecules in random haplotypes can have overlaps up to 28% in their peptide repertoires (see Figure 1C and results not shown), the distribution of the overlaps is not significantly different from the distribution given in Figure 1A. The set of random haplotypes was generated 100 times, and in none of these cases was the distribution significantly different from the one given in Figure 1A (using a Kolmogorov–Smirnov test). Finally, we calculated a weighted peptide repertoire overlap for the random haplotypes by assuming that the frequency of a random haplotype is simply the multiplication of the frequency of their HLA-A and HLA-B alleles (i.e., assuming a complete lack of linkage disequilibrium). Again, the distribution of weighted overlap of random haplotypes (Figure 1D) is not different from that of the real haplotypes (Figure 1B). Taken together, these results suggest that HLA-A and -B in pairs in general have distinct peptide binding preferences, and that a small overlap is not a unique property of the HLA molecules having a strong linkage disequilibrium.

The overlap distribution presented in Figure 1 seems to be in contradiction with earlier results, which estimated cross loci peptide overlaps of 23–44% (7, 9). However, this overlap was estimated at the population level, i.e., these percentages reflect the fraction of the peptide repertoire of an HLA-A molecule which is also expected to be presented by at least one HLA-B molecule in the population (and vice versa). Within an individual having maximally two different HLA-A and HLA-B molecules, the overlaps should remain much lower than the population based overlaps. In addition, one needs to realize that the low overlaps presented in Figure 1 depend on the threshold used to define the peptide repertoire of an HLA molecule. When we use a higher threshold of 2 or 5% the average overlap increases to 2.3 and 5.9%, respectively (with the standard threshold of 1%, the average overlap was 1.1%, see above). However, the choice of the threshold hardly affects our main result, namely that the HLA-A and -B pairs that are in a linkage disequilibrium do not have more distinct binding motifs than random HLA-A/B pairs (Figure 1).

## Conclusion

We hypothesized that it should be advantageous to have HLA molecules with distinct binding specificities combined in a haplotype, because during a viral infection such haplotypes would give an individual a larger epitope repertoire than haplotypes containing HLA molecules with similar binding motifs during a viral infection. To test this hypothesis, we used the high-resolution data available in the NMDP database on haplotype frequencies, and employed state of the art peptide-HLA binding prediction tools. We find that for all the haplotypes we could reliably identify in the US population, their HLA-A and HLA-B molecules present largely distinct set of peptides (Figure 1A). However, this turned out to be a generic property of HLA-A and HLA-B molecules: when we compared random HLA-A and -B pairs we find a very similar distribution of the presented peptide overlaps (Figure 1C). Moreover, there is no evidence for selection as there is no correlation between the population frequency of the HLA-A-B haplotypes and the overlap in the peptide repertoires of their HLA-A and HLA-B molecules. Taken together, these results suggest the complementarity of binding motifs is a general property of HLA-A and HLA-B molecules, and that complementarity does not affect the HLA haplotype composition. We were not able to specifically test the effect of complimentary binding motifs in the context of autoimmunity and cancer, as for both cases it remains unclear which set of human proteins should be taken as possible auto antigens. Complimentary binding motifs are expected to increase the number of potential self antigens, which could increase the risk of autoimmunity. Finally, the frequency of an HLA haplotype is determined by complex interactions with many different factors, one example is the correlation between birth weight and particular haplotypes (31). Our results suggest that complimentary binding motifs of HLA molecules during viral infections play a minor role, if any, compared to these other factors in the evolution of HLA haplotype frequencies.

## Conflict of Interest Statement

The authors declare that the research was conducted in the absence of any commercial or financial relationships that could be construed as a potential conflict of interest.

## Supplementary Material

The Supplementary Material for this article can be found online at http://www.frontiersin.org/Journal/10.3389/fimmu.2013.00374/abstract

## Footnotes

^1^www.allelefrequencies.net

^2^www.nmdp.org

^3^http://bioinformatics.nmdp.org

^4^www.ebi.ac.uk

## References

[1] Carrington M Nelson GW Martin MP Kissner T Vlahov D Goedert JJ HLA and HIV-1: heterozygote advantage and B*35-Cw*04 disadvantage. Science (1999) 283:1748–52 10.1126/science.283.5408.1748 10073943

[2] Apanius V Penn D Slev PR Ruff LR Potts WK The nature of selection on the major histocompatibility complex. Crit Rev Immunol (1997) 17:179–224 10.1615/CritRevImmunol.v17.i2.40 9094452

[3] Boehm T Zufall F MHC peptides and the sensory evaluation of genotype. Trends Neurosci (2006) 29:100–7 10.1016/j.tins.2005.11.006 16337283

[4] De Boer RJ Borghans JAM van Boven M Kesmir C Weissing FJ Heterozygote advantage fails to explain the high degree of polymorphism of the MHC. Immunogenetics (2004) 55:725–31 10.1007/s00251-003-0629-y 14722686

[5] O’Sullivan D Arrhenius T Sidney J Del Guercio MF Albertson M Wall M On the interaction of promiscuous antigenic peptides with different DR alleles. Identification of common structural motifs. J Immunol (1991) 1950(147):2663–9 1717570

[6] Axelsson-Robertson R Weichold F Sizemore D Wulf M Skeiky YA Sadoff J Extensive major histocompatibility complex class I binding promiscuity for *Mycobacterium tuberculosis* TB10.4 peptides and immune dominance of human leucocyte antigen (HLA)-B*0702 and HLA-B*0801 alleles in TB10.4 CD8 T-cell responses. Immunology (2010) 129:496–505 10.1111/j.1365-2567.2009.03201.x 20002212 PMC2842496

[7] Frahm N Yusim K Suscovich TJ Adams S Sidney J Hraber P Extensive HLA class I allele promiscuity among viral CTL epitopes. Eur J Immunol (2007) 37:2419–33 10.1002/eji.200737365 17705138 PMC2628559

[8] Nakagawa M Kim KH Gillam TM Moscicki A-B HLA class I binding promiscuity of the CD8 T-cell epitopes of human papillomavirus type 16 E6 protein. J Virol (2007) 81:1412–23 10.1128/JVI.01768-06 17108051 PMC1797519

[9] Rao X Hoof I Costa AI van Baarle D Kesmir C HLA class I allele promiscuity revisited. Immunogenetics (2011) 63:691–701 10.1007/s00251-011-0552-6 21695550 PMC3190086

[10] Carrington M Recombination within the human MHC. Immunol Rev (1999) 167:245–56 10.1111/j.1600-065X.1999.tb01397.x 10319266

[11] Begovich AB McClure GR Suraj VC Helmuth RC Fildes N Bugawan TL Polymorphism, recombination, and linkage disequilibrium within the HLA class II region. J Immunol (1992) 1950(148):249–58 1727870

[12] Smith WP Vu Q Li SS Hansen JA Zhao LP Geraghty DE Toward understanding MHC disease associations: partial resequencing of 46 distinct HLA haplotypes. Genomics (2006) 87:561–71 10.1016/j.ygeno.2005.11.020 16434165

[13] Madeleine MM Johnson LG Smith AG Hansen JA Nisperos BB Li S Comprehensive analysis of HLA-A, HLA-B, HLA-C, HLA-DRB1, and HLA-DQB1 loci and squamous cell cervical cancer risk. Cancer Res (2008) 68:3532–9 10.1158/0008-5472.CAN-07-6471 18451182 PMC2662593

[14] Albanidou-Farmaki E Deligiannidis A Markopoulos AK Katsares V Farmakis K Parapanissiou E HLA haplotypes in recurrent aphthous stomatitis: a mode of inheritance? Int J Immunogenet (2008) 35:427–32 10.1111/j.1744-313X.2008.00801.x 19046300

[15] Hoof I Peters B Sidney J Pedersen LE Sette A Lund O NetMHCpan, a method for MHC class I binding prediction beyond humans. Immunogenetics (2009) 61:1–13 10.1007/s00251-008-0341-z 19002680 PMC3319061

[16] Maiers M Gragert L Klitz W High-resolution HLA alleles and haplotypes in the United States population. Hum Immunol (2007) 68:779–88 10.1016/j.humimm.2007.04.005 17869653

[17] Kollman C Maiers M Gragert L Müller C Setterholm M Oudshoorn M Estimation of HLA-A, -B, -DRB1 haplotype frequencies using mixed resolution data from a National Registry with selective retyping of volunteers. Hum Immunol (2007) 68:950–8 10.1016/j.humimm.2007.10.009 18191722

[18] Long JC Williams RC Urbanek M An E-M algorithm and testing strategy for multiple-locus haplotypes. Am J Hum Genet (1995) 56:799–810 7887436 PMC1801177

[19] Excoffier L Slatkin M Maximum-likelihood estimation of molecular haplotype frequencies in a diploid population. Mol Biol Evol (1995) 12:921–7 7476138 10.1093/oxfordjournals.molbev.a040269

[20] MacNamara A Kadolsky U Bangham CR Asquith B T-cell epitope prediction: rescaling can mask biological variation between MHC molecules. PLoS Comput Biol (2009) 5:e1000327 10.1371/journal.pcbi.1000327 19300484 PMC2650421

[21] Buus S Lauemoller SL Worning P Kesmir C Frimurer T Corbet S Sensitive quantitative predictions of peptide-MHC binding by a “Query by Committee” artificial neural network approach. Tissue Antigens (2003) 62:378–84 10.1034/j.1399-0039.2003.00112.x 14617044

[22] Nielsen M Lundegaard C Worning P Lauemoller SL Lamberth K Buus S Reliable prediction of T-cell epitopes using neural networks with novel sequence representations. Protein Sci (2003) 12:1007–17 10.1110/ps.0239403 12717023 PMC2323871

[23] Crawford DC Nickerson DA Definition and clinical importance of haplotypes. Annu Rev Med (2005) 56:303–20 10.1146/annurev.med.56.082103.104540 15660514

[24] Yan H Papadopoulos N Marra G Perrera C Jiricny J Boland CR Conversion of diploidy to haploidy. Nature (2000) 403:723–4 10.1038/35002251 10693791

[25] Douglas JA Boehnke M Gillanders E Trent JM Gruber SB Experimentally-derived haplotypes substantially increase the efficiency of linkage disequilibrium studies. Nat Genet (2001) 28:361–4 10.1038/ng582 11443299

[26] Niu T Algorithms for inferring haplotypes. Genet Epidemiol (2004) 27:334–47 10.1002/gepi.20024 15368348

[27] Bettencourt BF Santos MR Fialho RN Couto AR Peixoto MJ Pinheiro JP Evaluation of two methods for computational HLA haplotypes inference using a real dataset. BMC Bioinformatics (2008) 9:68 10.1186/1471-2105-9-68 18230173 PMC2268655

[28] Castelli EC Mendes-Junior CT Veiga-Castelli LC Pereira NF Petzl-Erler ML Donadi EA Evaluation of computational methods for the reconstruction of HLA haplotypes. Tissue Antigens (2010) 76:459–66 10.1111/j.1399-0039.2010.01539.x 20670352

[29] Middleton D Menchaca L Rood H Komerofsky R New allele frequency database: http://www.allelefrequencies.net. Tissue Antigens (2003) 61:403–7 10.1034/j.1399-0039.2003.00062.x 12753660

[30] Nielsen M Lundegaard C Blicher T Lamberth K Harndahl M Justesen S NetMHCpan, a method for quantitative predictions of peptide binding to any HLA-A and -B locus protein of known sequence. PLoS One (2007) 2:e796 10.1371/journal.pone.0000796 17726526 PMC1949492

[31] Capittini C Pasi A Bergamaschi P Tinelli C De Silvestri A Mercati MP HLA haplotypes and birth weight variation: is your future going to be light or heavy? Tissue Antigens (2009) 74:156–63 10.1111/j.1399-0039.2009.01282.x 19500315

