# Supplementary material for: Complementarity of Binding Motifs is a General Property of HLA-A and HLA-B Molecules and Does Not Seem to Effect HLA Haplotype Composition
Source: Front Immunol. 2013 Nov 14;4:374. doi: 10.3389/fimmu.2013.00374 (PMC3827838; doi:10.3389/fimmu.2013.00374)
Supplement: Supplementary file 1 [file 63526_Kesmir_DataSheet1.DOCX]

## Supplementary Materials

Table S1

List of all reliable haplotypes (with a positive LD and significantly different than zero, p<0.01) in four ethnic groups in US from NMDP database. O_500_ and O_top_ correspond to the overlaps estimated using two different approaches to define the peptide repertoire of HLA-A and HLA-B molecules in a haplotype (see Methods). The last four columns give the population frequencies in European, African, Asian, and Hispanic Americans, respectively.

| Haplotype | O_500_ | O_top_ | EUR | AFA | API | HIS |
| --- | --- | --- | --- | --- | --- | --- |
| A0101_B0801 | 0 | 0 | 0.095 | 0.015 | 0.0041 | 0.022 |
| A0101_B1517 | 0.049 | 0.12 | - | - | 0.0039 | - |
| A0101_B3701 | 0 | 0 | 0.0082 | 0.0021 | 0.0087 | - |
| A0101_B5701 | 0.034 | 0.053 | 0.021 | 0.0026 | 0.014 | 0.0074 |
| A0102_B4901 | 0 | 0.00088 | - | 0.0029 | - | - |
| A0201_B1501 | 0.077 | 0.026 | 0.037 | 0.0047 | - | - |
| A0201_B1511 | 0.027 | 0.017 | - | - | 0.0038 | - |
| A0201_B1515 | 0.031 | 0.025 | - | - | - | 0.005 |
| A0201_B2705 | 0.003 | 0.0044 | 0.015 | - | - | - |
| A0201_B3512 | 0.0004 | 0.014 | - | - | - | 0.011 |
| A0201_B4001 | 0.0062 | 0.0053 | 0.028 | 0.005 | - | - |
| A0201_B4402 | 0 | 0 | 0.057 | 0.013 | - | 0.019 |
| A0201_B4501 | 0.0021 | 0 | - | 0.017 | - | - |
| A0201_B5101 | 0.0012 | 0.012 | 0.011 | 0.0061 | - | 0.022 |
| A0201_B5401 | 0.0022 | 0.011 | - | - | 0.0079 | - |
| A0202_B1516 | 0.1 | 0.0071 | - | - | - | 0.0024 |
| A0202_B4101 | 0.0028 | 0.00088 | 0.00057 | - | - | - |
| A0202_B5301 | 0.0035 | 0.00088 | - | 0.01 | - | - |
| A0203_B3802 | 0 | 0.028 | - | - | 0.012 | - |
| A0205_B4901 | 0.0046 | 0.022 | 0.0014 | - | - | 0.0029 |
| A0205_B5001 | 0.021 | 0.017 | 0.0036 | - | 0.0028 | - |
| A0205_B5801 | 0.021 | 0.0035 | 0.001 | 0.0039 | - | 0.0025 |
| A0206_B2705 | 0.0028 | 0.0062 | 0.00068 | - | - | - |
| A0206_B3905 | 0.024 | 0.061 | - | - | - | 0.0064 |
| A0206_B4002 | 0.053 | 0.038 | - | - | - | 0.0085 |
| A0206_B4801 | 0.022 | 0.086 | 0.00044 | - | - | 0.0052 |
| A0206_B5101 | 0.0026 | 0.019 | - | - | 0.0094 | - |
| A0206_B5901 | 0.015 | 0.034 | - | - | 0.0035 | - |
| A0207_B4601 | 0 | 0.022 | - | - | 0.033 | - |
| A0211_B4006 | 0.023 | 0.0071 | - | - | 0.0047 | - |
| A0301_B0702 | 0 | 0 | 0.06 | 0.017 | - | 0.019 |
| A0301_B1402 | 0 | 0 | 0.0072 | - | - | - |
| A0301_B3501 | 0.014 | 0.0071 | 0.011 | 0.012 | 0.0049 | 0.011 |
| A0301_B3503 | 0 | 0.0026 | 0.0031 | - | - | - |
| A0301_B4701 | 0.0025 | 0.00088 | 0.0021 | - | - | - |
| A1101_B1301 | 0 | 0 | - | - | 0.012 | - |
| A1101_B1502 | 0.034 | 0.0088 | - | - | 0.011 | - |
| A1101_B2705 | 0.003 | 0.0035 | - | - | - | 0.0058 |
| A1101_B3501 | 0.021 | 0.0044 | 0.017 | 0.0036 | - | 0.0071 |
| A1101_B3901 | 0 | 0 | - | - | 0.0065 | - |
| A1101_B4001 | 0 | 0 | - | - | 0.024 | - |
| A1101_B5101 | 0 | 0 | 0.0043 | - | - | - |
| A1101_B5201 | 0 | 0 | 0.0028 | - | - | - |
| A1101_B5501 | 0 | 0 | 0.0058 | 0.0021 | - | - |
| A1102_B2704 | 0.0015 | 0.0044 | - | - | 0.0034 | - |
| A2301_B1503 | 0.034 | 0.035 | - | 0.015 | - | 0.003 |
| A2301_B4403 | 0.0074 | 0.0097 | 0.0086 | - | - | 0.0097 |
| A2301_B4501 | 0.0056 | 0.0035 | - | 0.0097 | - | 0.0033 |
| A2301_B4901 | 0.0068 | 0.011 | 0.0037 | - | - | 0.0038 |
| A2402_B1501 | 0.0096 | 0.022 | 0.0095 | - | - | - |
| A2402_B1507 | 0.011 | 0.018 | 0.00063 | - | - | - |
| A2402_B3502 | 0 | 0.0035 | 0.0049 | - | - | 0.0062 |
| A2402_B3508 | 0.00081 | 0.0018 | 0.0012 | - | - | - |
| A2402_B3906 | 0.0029 | 0.0053 | 0.0027 | 0.0015 | - | 0.012 |
| A2402_B4002 | 0.011 | 0.012 | - | - | 0.012 | 0.012 |
| A2402_B4005 | 0.0097 | 0.012 | - | - | - | 0.0034 |
| A2402_B4801 | 0.0038 | 0.015 | - | - | 0.0092 | - |
| A2402_B5401 | 0 | 0 | - | - | 0.012 | - |
| A2402_B5501 | 0 | 0.00088 | 0.0032 | - | - | - |
| A2403_B1801 | 0.011 | 0.0035 | - | - | 0.0011 | - |
| A2407_B3505 | 0.0013 | 0.0044 | - | - | 0.0064 | - |
| A2417_B1502 | 0.0049 | 0.023 | - | - | 0.0031 | - |
| A2501_B1801 | 0.0072 | 0.0071 | 0.011 | 0.0029 | - | 0.0048 |
| A2501_B3901 | 0.0035 | 0.0044 | 0.00089 | - | - | - |
| A2601_B0801 | 0.012 | 0.012 | - | 0.0031 | 0.0052 | - |
| A2601_B1401 | 0 | 0.0053 | 0.00094 | - | - | - |
| A2601_B2705 | 0 | 0 | 0.0021 | - | - | - |
| A2601_B3801 | 0 | 0.0035 | 0.011 | - | - | 0.0085 |
| A2601_B4501 | 0 | 0 | 0.00082 | - | - | - |
| A2601_B5501 | 0 | 0.012 | 0.0016 | - | - | - |
| A2901_B0705 | 0.0013 | 0.00088 | 0.001 | - | 0.012 | - |
| A2902_B4403 | 0.0044 | 0.0018 | 0.024 | 0.011 | - | 0.025 |
| A2902_B4404 | 0.0047 | 0.0018 | 0.00057 | - | - | - |
| A2902_B4501 | 0.00067 | 0.00088 | 0.0021 | - | - | 0.0035 |
| A2902_B4901 | 0 | 0.0018 | - | 0.0036 | - | - |
| A3001_B1302 | 0.00035 | 0.0035 | 0.011 | - | 0.019 | 0.0053 |
| A3001_B4201 | 0.0024 | 0.021 | - | 0.021 | - | 0.004 |
| A3001_B4202 | 0.014 | 0.023 | - | 0.0058 | - | - |
| A3002_B1402 | 0 | 0 | - | 0.0044 | - | - |
| A3002_B1801 | 0.0065 | 0.0053 | 0.0058 | - | - | 0.01 |
| A3002_B5703 | 0.0041 | 0.012 | - | 0.0053 | - | - |
| A3004_B1401 | 0 | 0.0026 | - | - | 0.0028 | - |
| A3101_B2705 | 0.0068 | 0.0044 | 0.0018 | - | - | - |
| A3101_B4001 | 0 | 0 | 0.0088 | 0.0027 | - | - |
| A3101_B5101 | 0 | 0 | 0.0025 | - | 0.0072 | - |
| A3101_B5102 | 0.00044 | 0 | - | - | - | 0.0028 |
| A3201_B1401 | 0 | 0.021 | 0.0038 | - | - | - |
| A3201_B4002 | 0.034 | 0.011 | 0.0025 | - | - | - |
| A3201_B4402 | 0 | 0.0018 | 0.0041 | - | - | - |
| A3201_B8101 | 0.0005 | 0.024 | - | 0.0022 | - | - |
| A3301_B1402 | 0 | 0 | 0.0093 | 0.003 | - | 0.015 |
| A3301_B7801 | 0.001 | 0 | - | 0.0049 | - | - |
| A3303_B1516 | 0.00035 | 0 | - | 0.0055 | - | - |
| A3303_B4403 | 0 | 0 | - | - | 0.029 | - |
| A3303_B5301 | 0 | 0 | - | 0.013 | - | - |
| A3303_B5801 | 0 | 0 | 0.00076 | - | 0.045 | - |
| A3401_B1521 | 0.061 | 0.071 | - | - | 0.0011 | - |
| A3401_B1535 | 0.031 | 0.045 | - | - | 0.0036 | - |
| A3402_B3501 | 0.033 | 0.016 | - | 0.0056 | - | - |
| A3402_B4403 | 0 | 0 | - | 0.0078 | - | - |
| A3601_B5301 | 0.028 | 0.035 | - | 0.015 | - | 0.002 |
| A6601_B4102 | 0 | 0 | 0.0019 | - | - | 0.0025 |
| A6601_B5802 | 0 | 0.013 | - | 0.0043 | - | - |
| A6602_B5801 | 0.017 | 0.011 | - | 0.0037 | - | - |
| A6801_B3503 | 0 | 0 | 0.0023 | - | - | - |
| A6801_B4001 | 0 | 0 | 0.0036 | - | - | - |
| A6801_B4002 | 0.0003 | 0 | - | - | - | 0.0078 |
| A6801_B4402 | 0 | 0 | 0.0056 | - | - | - |
| A6801_B4801 | 0 | 0 | - | - | - | 0.0058 |
| A6801_B5101 | 0 | 0 | 0.0028 | - | - | - |
| A6801_B5201 | 0 | 0 | - | - | 0.0041 | - |
| A6801_B5802 | 0 | 0.00088 | - | 0.012 | - | - |
| A6802_B1402 | 0 | 0.013 | 0.0057 | - | - | 0.0056 |
| A6802_B1510 | 0.007 | 0.013 | - | 0.012 | - | 0.0025 |
| A6802_B5301 | 0.02 | 0.014 | 0.002 | 0.014 | - | 0.0052 |
| A6803_B3905 | 0.00063 | 0.00088 | - | - | - | 0.0066 |
| A6901_B5501 | 0.012 | 0.028 | 0.00064 | - | - | 0.0025 |
| A7401_B1503 | 0.0019 | 0 | - | 0.016 | - | 0.0025 |
| A7401_B5703 | 0.0022 | 0 | - | 0.0051 | - | - |
| A8001_B1801 | 0.0063 | 0.0062 | - | 0.0021 | - | - |

Table S2. List of human viral proteomes used in this study.

| VIRUS | |
| --- | --- |
| Human immunodeficiency virus 1 | X01762 |
| Dengue virus 1 | U88536 |
| Reston Ebola virus | AB050936 |
| Hepatitis A virus | M14707 |
| Hepatitis B virus | X51970 |
| Hepatitis C virus | AJ132997 |
| Human T-lymphotropic virus 1 | D13784 |
| Influenza A virus (A/Goose /Guangdong/1/96(H5N1)) segment 1-8 | AF144300-AF144307 |
| Measles virus | K01711 |
| Mumps virus | AB040874 |
| H-1 parvovirus | X01457 |
| Human poliovirus 1 | AJ132961 |
| Rabies virus | M31046 |
| Human respiratory syncytial virus | AF013254 |
| Rubella virus | AF188704 |
| Sendai virus | M69046 |
| Yellow fever virus | X03700 |

**Figure S1:**

The distribution of the presented peptide overlap (given in percentage) by HLA-A and HLA-B molecules belonging to the same haplotype using a much larger set of mammalian viruses (n=904) than the ones listed in Table S2.
